# Supplementary material for: Vti1a/b regulate synaptic vesicle and dense core vesicle secretion via protein sorting at the Golgi
Source: Nat Commun. 2018 Aug 24;9:3421. doi: 10.1038/s41467-018-05699-z (PMC6109172; doi:10.1038/s41467-018-05699-z)
Supplement: Supplementary file 1 — Supplementary Information [file 41467_2018_5699_MOESM1_ESM.pdf]

SUPPLEMENTARY INFORMATION

The supplementary information contains 12 supplementary figures (including legends) and one supplementary table

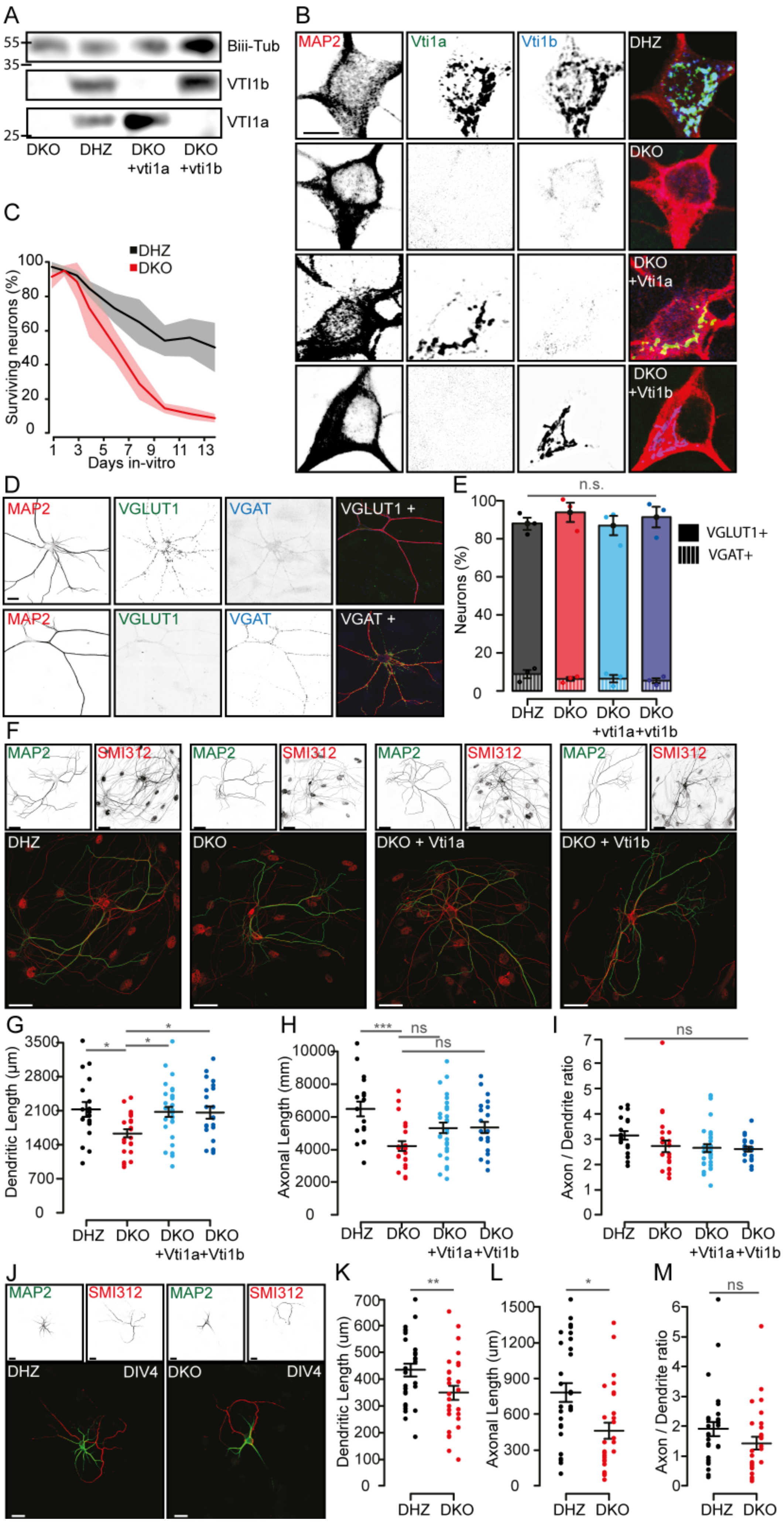

Supplementary Figure 1: Vti1a/b deficient neurons are smaller and show increased cell death. (A) WB of whole neuron lysates of DHZ, DKO or DKO neurons expressing Vti1a or Vti1b. (B) Representative examples of DIV-14 neurons immunostained for MAP2, Vti1a and Vti1b. (C) Survival plot of DHZ and DKO neurons shows that from DIV3 to DIV10 cell death in DKO (slope of 10.57±1.58) is faster than DHZ neurons (slope of 5.25±1.59). (D) Representative example of glutamatergic (top) and GABAergic (bottom) neurons stained for MAP2 and the glutamatergic and GABergic markers VGLUT1 and VGAT, respectively. (E) The percentage of glutamatergic neurons (Solid bars; DHZ: 78.89±3.19; DKO: 87.51±3.19; DKO+Vti1a: 80.35 + 5.19; DKO+Vti1b: 85.69±5.42%) and GABAergic neurons remains similar (striped bars; DHZ: 11.79±2.86; DKO: 8.42±1.28; DKO+1a: 8.52 + 2.61; DKO+1b: 7.19±1.69%). N=3 cultures; one-Way ANOVA. A small percentage of neurons negative for VGAT and VGLUT1 were observed in all groups. (F) Representative examples of single DIV-14 neurons immunostained for the dendritic marker MAP2 and the axonal marker SMI312. (G) Shorter dendritic length in DKO neurons (DHZ: 2128.16±153.98, n=18; DKO: 1632.20±84.06, n=24; DKO+Vti1a: 2072.44±100.22, n=31; DKO+Vti1b: 2061.26±124.04 µm,

(Legend continues on next page)

n=22; one-way ANOVA). (H) Shorter axonal length in DKO neurons (DHZ: 6473.90 ±454.80, n=18; DKO: 4211.42±291.66, n=24; DKO+Vti1a: 5315.35±321.69, n=31; DKO+Vti1b: 5336.37±345.97 µm, n=22; one-way ANOVA). (I) Similar axon-to-dendrite ratio between groups (DHZ: 3.13±0.18, n=18; DKO: 2.72±0.24, n=24; DKO+Vti1a: 2.64±0.16, n=31; DKO+Vti1b: 2.61±0.10, n=22; Kruskal-Wallis). (J) Representative examples of single DIV4 neurons immunostained for MAP2 and SMI312. (K) Reduced dendritic length at DIV4 in DKO neurons (DKO: 460.89±66.32, n=24; DHZ: 783.35±79.95 µm, n=29; Wilcoxon). (L) Decreased axonal length at DIV4 in DKO neurons (DKO: 348.66±25.90, n=24; DHZ: 433.98±23.86 µm, n=29; Student's t-test). (M) Similar axon-to-dendrite ratio at DIV4 (DKO: 1.44±0.22, n=24; DHZ: 1.92±0.24, n=29; Wilcoxon). Bars show mean±SEM. Scatterplots and columns represent individual neurons and independent litters, respectively.*$p < 0.05$; **$p < 0.01$; ***$p < 0.001$. Note that SMI312 immunoreacts with neuronal and glial nuclei. Scale bars=10 µm (B), 20um (D), 40 µm (F), 20 µm (J).

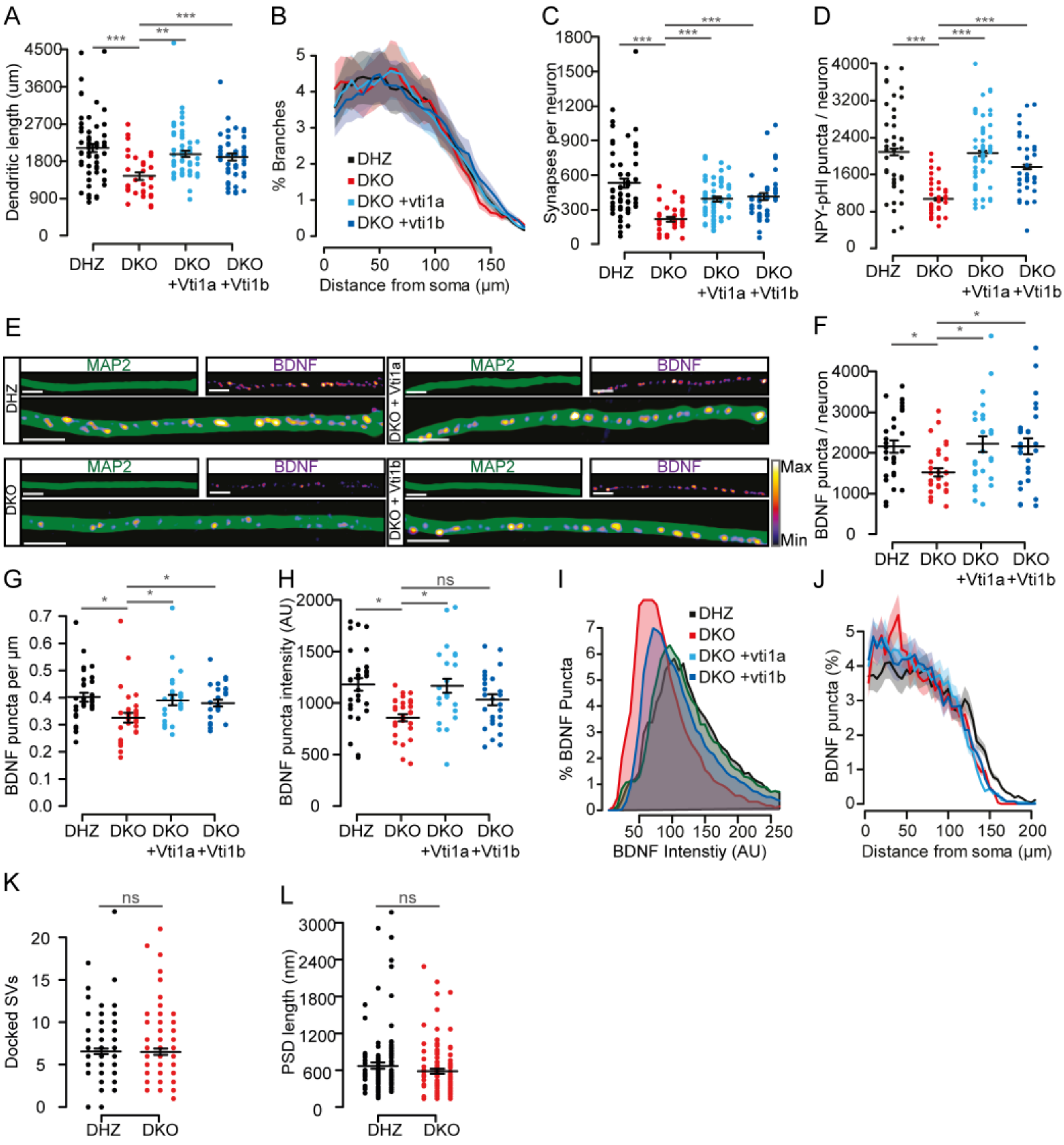

Supplementary Figure 2: Vti1a/b deficient neurons are smaller, make fewer synapses and contain less DCVs. (A) Reduced dendritic length in DKO neurons (DHZ: 2121.17±106.58; DKO: 1451.39±92.63; DKO+Vti1a: 1973.86±76.27; DKO+VTI1b: 1900.90±85.04 μm; Kruskal-Wallis). (B) Sholl analysis showing similar distribution of dendritic branches. (C) Reduced synapse number in DKO neurons (DHZ: 531.40±40.79; DKO: 218.5±21.11; DKO+Vti1a: 393.68±21.66; DKO+Vti1b: 414±31.13 synapses/ neuron; Kruskal-Wallis). (D) Reduced number of NPY-pHluorin puncta per neuron in DKO neurons (DHZ: 2087.6±71.0, n=42; DKO: 1062.2±30.2, n=37; DKO+Vti1a: 2056.2±54.8, n=59; DKO+Vti1b: 1759.3±50.0 puncta, n=34; Kruskal-Wallis). (E) Representative examples of single DIV-14 neurons stained for MAP2 and BDNF. Intensity color-code used for BDNF. Scale bar=5μm. (F) Rerduced number of BDNF puncta in DKO neurons (DHZ: 2154.5±147.3, n=30; DKO: 1525.2±108.0, n=31, DKO+Vti1a: 2217.8±198.6, n=26; DKO+Vti1b: 2164.0±193.0 puncta per neuron, n=27; one-way ANOVA). (G) Reduced BDNF puncta density in DKO neurons (DHZ: 0.40±0.02, n=30; DKO: 0.33±0.03, n=31, DKO+Vti1a: 0.39±0.02, n=26; DKO+Vti1b: 0.38±0.01 puncta/ μm neurite, n=27; Kruskal-Wallis). (H) Decreased BDNF puncta intensity in DKO neurons (DHZ: 117.10±6.21, n=30; DKO: 85.23±3.31, n=31, DKO+Vti1a: 115.80±6.94, n=26; DKO+Vti1b: 102.67±5.46 AU, n=27; Kruskal-Wallis). (I) Histogram of all BDNF puncta intensity. (J) Sholl analysis of BDNF puncta. (K) Docked SVs per synaptic micrograph (DHZ:6.55±0.32; n=122; DKO: 6.53±0.35 SVs; n=119; Mann-Whitney). (L) Postsynaptic density length (DHZ: 675.18±47.46; n=122; DKO: 586.33±36.49nm; n=119; Mann-Whitney U-test).Bars show mean±SEM. Scatterplots and columns represent individual neurons and independent litters, respectively.*p<0.05; **p<0.01; ***p<0.001.

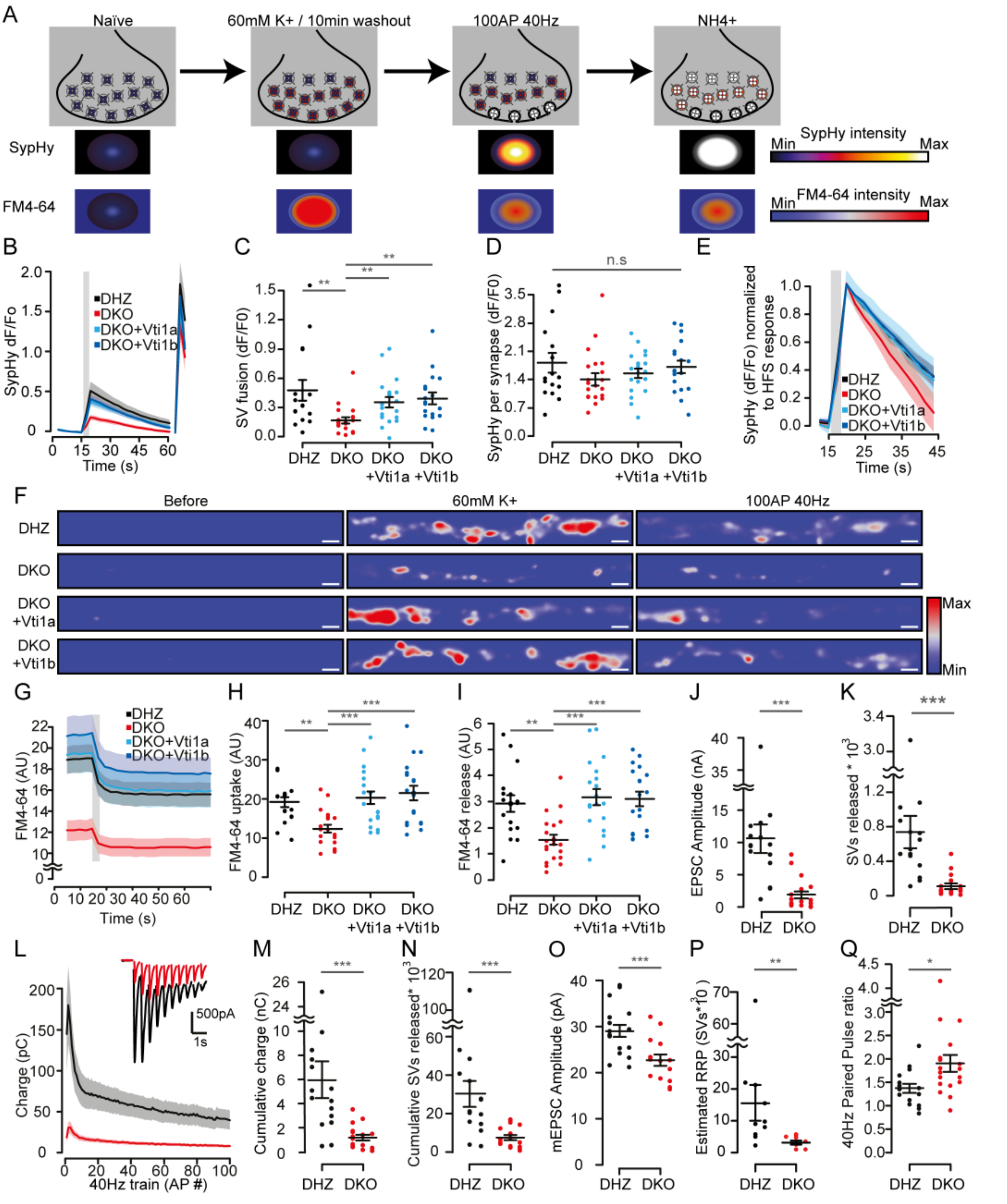

Supplementary Figure 3: SV secretion is severely reduced in Vti1a/b deficient neurons. (A) Cartoon showing experimental design. Initially, SypHy is quenched (Naïve). Recycling SVs are labelled by 60s-incubation with FM4-64 in 60mM K^+, followed by a 10min washout. High frequency stimulation (HFS; 100AP, 40Hz) triggers SV fusion witnessed as increased SypHy and decreased FM4-64 fluorescence intensity. Ultimately, NH_4^+ is superfused to visualize total SypHy fluorescence, used as estimation of the total SV pool. (B) Average SypHy traces in active synapses upon HFS (grey rectangle) and NH_4^+ superfusion (right traces). (C) Decreased SV fusion in DKO neurons (DHZ: 0.477±0.106, n=17; DKO: 0.165±0.031, n=21; DKO+Vti1a: 0.354±0.052, n=20; DKO+Vti1b: 0.393±0.059 AU, n=19; Kruskal-Wallis). (D) SypHy expression per synapse (DHZ: 1.82±0.24, n=17; DKO: 1.41±0.15, n=21; DKO+Vti1a: 1.56±0.12, n=20; DKO+Vti1b: 1.72±0.15 AU, n=19; one-way ANOVA). (E) Average trace of SV fusion normalized to the maximum response. (F) Representative examples of synapses before (left), after incubation with FM4-64 and 60 mM K^+ (middle) and after HFS (right). Intensity color-code used for SypHy representation

(Legend continues on next page)

(blue and red as lowest and highest, respectively). Scale bar=2μm. (G) Average FM4-64 traces showing initial SV loading, followed by release upon HFS (grey rectangle). (H) FM4-64 uptake (upon 60 mM K^+ incubation) is decreased in DKO neurons (DHZ: 19.00±1.29, n=17; DKO: 12.24±9.73, n=21; DKO+Vti1a: 20.10±1.57, n=20; DKO+Vti1b: 21.32±1.80 AU, n=19; one-way ANOVA). (I) FM4-64 release upon HFS is decreased in DKO neurons (DHZ: 2.91±3.09, n=17; DKO: 1.53±1.90, n=21; DKO+Vti1a: 3.15±3.02, n=20; DKO+Vti1b: 3.08±2.71 AU, n=19; one-way ANOVA). (J) Evoked EPSC amplitude is reduced in DKO neurons (DHZ: 10.70±2.23, n=15; DKO: 1.94±0.57 nA, n=18; Mann-Whitney). (K) The number of SVs released by one action potential (AP), calculated by charge transfer of the evoked response over mEPSC charge, is reduced in DKO neurons (DHZ: 734.31±187.01, n=15; DKO: 111.11±30.89 SVs, n=18; Mann-Whitney). (L) Evoked EPSC charge transfer per AP during a 100 AP at 40 Hz train stimulation. Inset shows representative traces of evoked release during the first ten APs. (M) The cumulative evoked EPSC charge transfer during HFS (100APs, 40 Hz) is reduced in DKO neurons (DHZ: 5.99±1.54, n=15; DKO: 1.23±0.23 nC, n=18; Mann-Whitney). (N) The cumulative number of SVs released by the train of 100 APs at 40 Hz, calculated by charge transfer over mEPSC charge, is reduced in DKO neurons (DHZ: 30328±6883, n=15; DKO: 7747±1334 SVs, n=18; Mann-Whitney). (O) The spontaneous mEPSC amplitude is reduced in DKO neurons (DHZ: 27.60±1.26, n=18; DKO: 21.61±1.15 pA, n=15, t-test). (P) The RRP size as number of SVs, estimated as sucrose-induced released charge over average mEPSC charge per neuron, is reduced in DKO neurons (DHZ: 6315.75±2069.43 SVs, n=11; DKO: 1422.58±308.80 SVs, n=10; Mann-Whitney). (Q) 40 Hz paired pulse ratio is increased in DKO neurons (DHZ: 1.38±0.10, n=15; DKO: 1.91±0.18%, n=18; Mann-Whitney). Bars show mean±SEM. Shaded regions indicate SEM. Scatterplots and columns represent individual neurons and independent litters, respectively. **p<0.01; ***p<0.001.

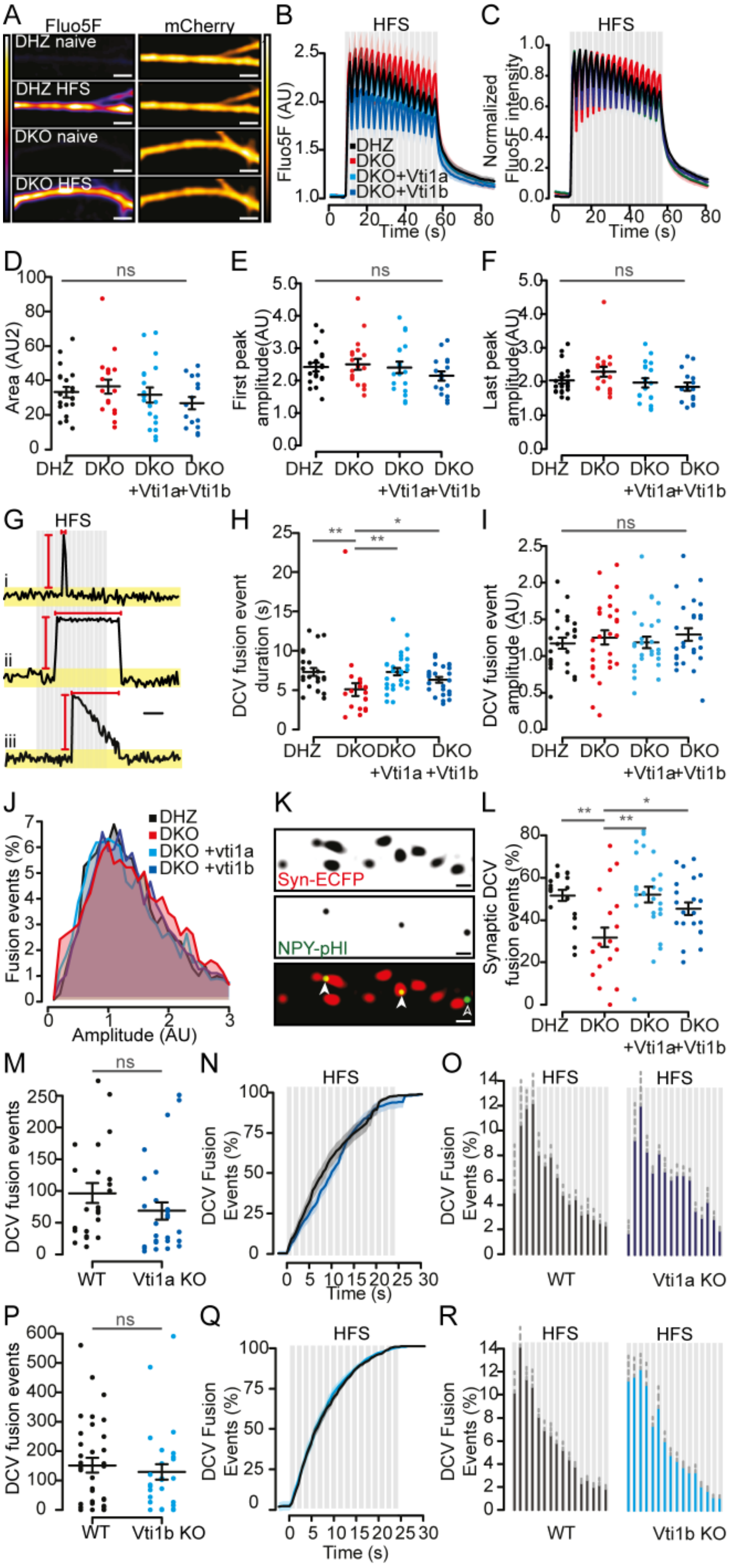

Supplementary Figure 4: Impaired Dense core vesicle secretion is in Vti1a/b deficient neurons. (A) Representative examples of single DIV-14 neurons expressing soluble mCherry (used as control for volume or pH changes) and loaded with the Fluo5F-AM, before and after high frequency stimulation (HFS, 16 trains, 50 APs at 50 Hz). Intensity color-code used for NPY-pHluorin and mCherry. (B-C) Raw (B) and normalized (C) intensity traces of Fluo5FM-loaded neurons during HFS. Shaded areas represent SEM. (D) Area of the Fluo5F response (DHZ: 33.27±3.06, n=20; DKO: 36.40±3.99, n=19; DKO+Vti1a: 31.52±4.32, n=19; DKO+Vti1b: 26.87±3.47 AU^2, n=16; Kruskal-Wallis). (E) Fluo5F amplitude after the first train of stimulation (DHZ: 2.42±0.13, n=20; DKO: 2.50±0.17, n=19; DKO+Vti1a: 2.40±0.18, n=19; DKO+Vti1b: 2.14±0.15 AU, n=16; One-Way ANOVA). (F) Fluo5F amplitude after the last train (DHZ: 2.03±0.10, n=20; DKO: 2.29±0.14, n=19; DKO+Vti1a: 1.97±0.14, n=19; DKO+Vti1b: 1.84±0.12 AU, n=16; Kruskal-Wallis). (G) Traces showing heterogeneity of DCV fusion events, characterized by rapid increase in fluorescence (amplitude, vertical red lines) and variable duration (horizontal red lines), determined as the time that fluorescence remains above baseline+3sd (yellow box). (H) DCV fusion events duration (DHZ: 7.24±0.50, n=24; DKO: 5.04±0.42, n=21; DKO+Vti1a: 7.27±0.47, n=27; DKO+Vti1b: 6.25±0.36s, n=25; Kruskal-Wallis). Neurons with<3 events were excluded. (I) Amplitude of DCV fusion events per neuron (DHZ: 1.18±0.08, n=24; DKO: 1.25±0.10, n=28; DKO+Vti1a:1.19±0.08, n=27; DKO+Vti1b: 1.30±0.09 AU, n=26; one-Way Anova). (J) Histogram showing similar distribution of the amplitude all DCV fusion events per group. (K) Example of synaptic (full arrowheads) and extrasynaptic (empty arrowheads) DCV fusion events. Synaptic events were detected as fusion occurring within a Synapsin-ECFP mask. (L) Percentage of synaptic DCV fusion events per neurons (DHZ: 51.57±2.54, DKO: 31.84±4.61, DKO+Vti1a: 51.99±3.69, DKO+Vti1b: 45.41±2.96%; Kruskal-Wallis). Neurons with<3 fusion events were not included. (M) DCV fusion events per neuron are not reduced in Vti1a KO neurons (WT: 87.78±14.17, n=23; Vti1a KO: 62.18±12.49 events, n=28; Mann-Whitney). (N, O) Normalized cumulative plot (N) and histogram (O) of the fraction of DCV fusion events. (P) DCV fusion events per neuron are not reduced in Vti1b KO neurons (WT: 168.09±27.87, n=34; Vti1b KO: 143.64±29.56 events, n=28; Mann-Whitney). (Q, R) Normalized cumulative plot (Q) and histogram (R) of the fraction of DCV fusion events. Bars show mean±SEM. Bullets and columns represent individual observations and litters, respectively. Grey bars = 16x50AP, 50Hz stimulation. *p<0.05, **p<0.01. Scale bars=5 µm (A), 5s (G), 1µm (K).

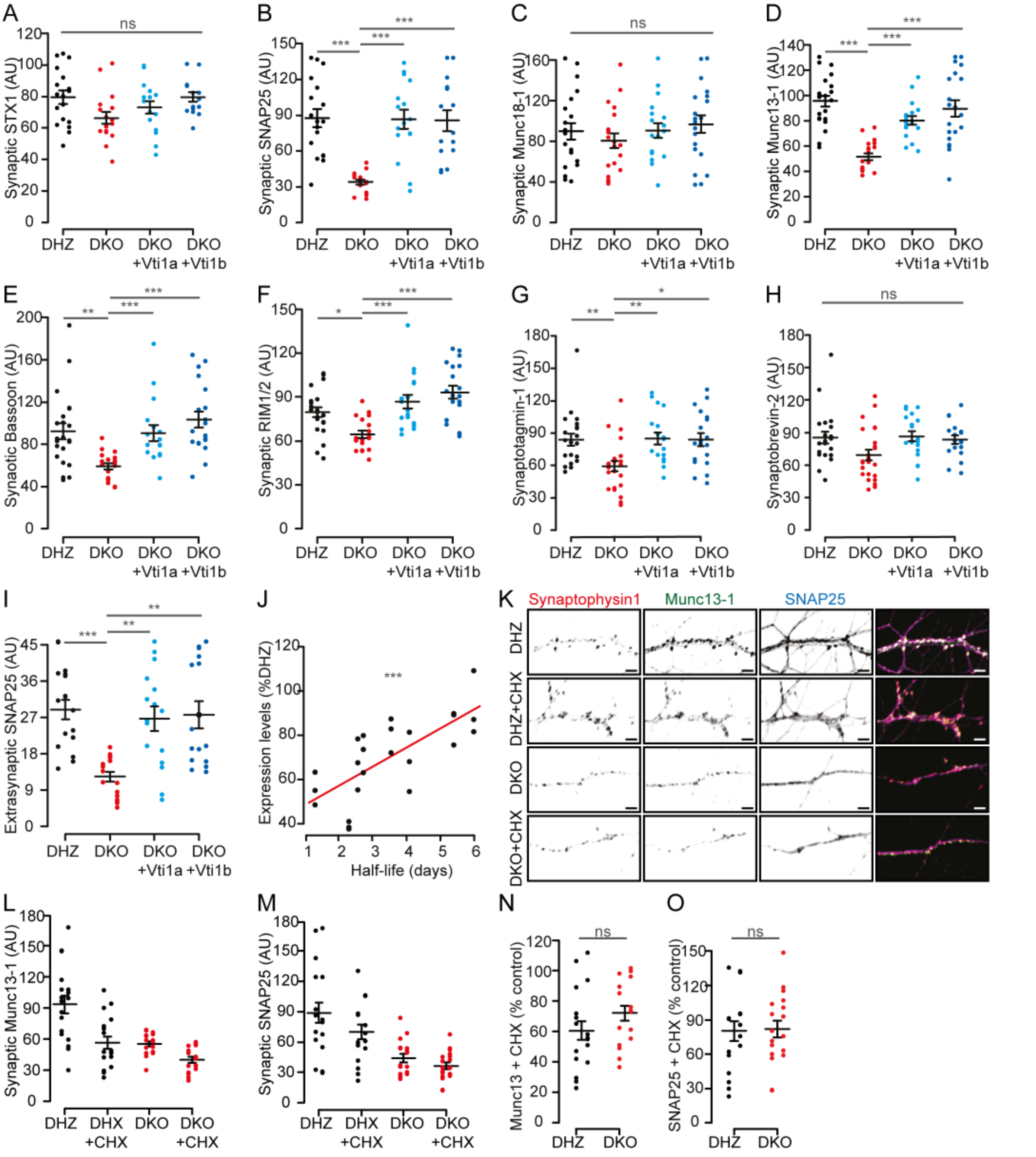

Supplementary Figure 5. Similar stability and reduced levels of exocytic proteins in Vti1a/b deficient neurons. (A) Synaptic levels of synaptic Syntaxin-1 (DHZ: 79.51±4.20, n=18; DKO=66.43±3.83, n=17; DKO+1a: 72.97±4.03, n=16; DKO +1b: 79.61±2.97 AU, n=15; One-Way ANOVA). (B) Synaptic levels of SNAP25 (DHZ: 83.73±7.21, n=18; DKO=32.40±1.93, n=17; DKO+1a: 82.53±7.65, n=16; DKO +1b: 81.60±8.32 AU, n=15; Kruskal-Wallis). (C) Synaptic levels of Munc18-1 (DHZ: 89.88±8.06, n=21; DKO=80.54±7.26, n=20; DKO+1a: 90.88±7.32, n=18; DKO +1b: 96.99±8.54 AU, n=20; one-way ANOVA). (D) Synaptic levels of Munc13-1 (DHZ: 98.45±4.49, n=21; DKO=53.11±2.72, n=20; DKO+1a: 82.81±3.64, n=18; DKO +1b: 92.44±6.53 AU, n=20; one-way ANOVA). (E) Synaptic levels of Bassoon (DHZ: 79.54±3.35, n=22; DKO=64.28±2.48, n=19; DKO+1a: 86.59±4.70, n=17; DKO +1b: 93.13±4.50 AU, n=19; Kruskal-Walis). (F) Synaptic levels of RIM1/2 (DHZ: 92.09±7.58, n=22; DKO=59.38±2.93, n=19; DKO+1a: 90.57±7.40, n=17; DKO +1b: 103.49±7.65 AU, n=19; Kruskal-Walis). (G) Synaptic levels of Synaptotagmin-1 (DHZ: 83.84±5.55, n=21; DKO=58.88±4.70, n=24; DKO+1a: 84.42±5.62, n=18; DKO +1b: 83.38±5.88 AU, n=19; Kruskal-Walis). (H) Synaptic levels of Synaptobrevin-2 (DHZ: 85.31±5455, n=21; DKO=68.04±4.91, n=24; DKO+1a: 86.04±7.76, n=18; DKO +1b: 82.83±3.80 AU, n=19; Kruskal-Walis). (I) Extrasynaptic levels of SNAP25 are also decreased in DKO neurons (DHZ: 28.94±2.40, n=18;

(Legend continues on next page)

DKO=12.35±1.20, n=17; DKO+1a: 26.69±3.02, n=16; DKO +1b: 27.63±3.40 AU, n=15; Kruskal-Wallis). (J) Positive correlation between the synaptic levels and protein half-life (from Cohen et al., 2013). R2=0.719; p<0.0001. Columns represent individual proteins, in triplicate. (K) Representative examples of DIV-14 single neurons incubated with cycloheximide (CHX) or vehicle for 24 hours and immunostained for Synaptophysin-1, Munc13-1 and SNAP25. (L) Synaptic levels of Munc13-1 (DHZ control: 93.62±8.55, n=18; DHZ+CHX=56.43±5.77, n=18; DKO naive: 55.33±2.52, n=17; DHZ+CHX: 40.04±2.75 AU, n=18). (M) Synaptic levels of SNAP25 (DHZ naive: 88.92±10.19, n=18; DHZ+CHX=69.93±7.04, n=18; DKO naive: 44.19±4.21, n=17; DHZ+CHX: 36.48±3.39 AU, n=18). (N) The remaining percentage of synaptic Munc13-1 levels after CHX incubation is similar between DKO and DHZ neurons (DHZ: 60.64±8.14, n=17; DKO: 72.10±4.90% of control, n=18; T-test). (O) The remaining percentage of synaptic SNAP25 levels remaining after CHX incubation is similar between DKO and DHZ neurons (DHZ: 80.36±8.59, n=17; DKO: 81.89±7.39% of control, n=18; t-test). Bars show mean±SEM. Scatterplots and columns represent individual neurons and independent litters, respectively.*p<0.05; **p<0.01; ***p<0.001. Scale bar=2 μm.

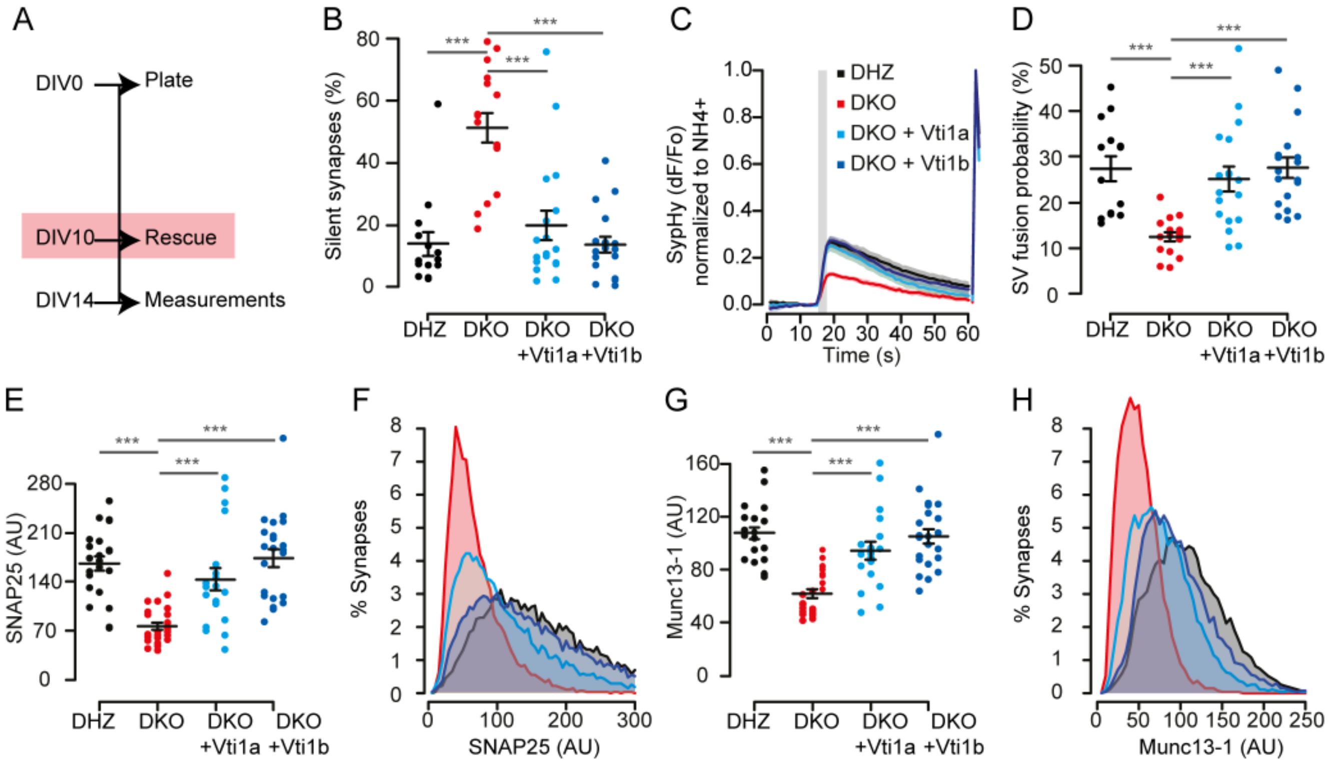

Supplementary Figure 6: Decreased SV fusion and levels of SNAP25 and Munc13-1 can be rescued by late expression of Vti1a or Vti1b. (A) Scheme of experiment, highlighting expression of Vti1a or Vti1b at DIV10. (B) Percentage of silent synapses (DHZ: 13.90±3.91 n=14; DKO: 51.41±4.80 n=16; DKO+Vti1a: 19.88±4.69% n=18, DKO+Vti1b: 13.64±2.55, n=13; ***p<0.001, Kruskal-Wallis). (C) Average SypHy traces of SV fusion upon HFS (100APs at 40 Hz; grey rectangle) normalized to the maximum intensity upon NH_4^+ superfusion (right traces). Shaded regions indicate SEM. (D) Quantification of (C). Fraction of SypHy-labelled SVs that fuse in responsive synapses (DHZ: 27.35±2.72 n=14; DKO: 12.55+1.03 n=16; DKO+Vti1a: 25.10±2.69 n=18, DKO+Vti1b: 27.53±2.23% n=13; ***p<0.01, Kruskal-Wallis). (E) The synaptic levels of SNAP25 are rescued by expression of Vti1a or Vti1b at DIV10 (DHZ: 166.06±10.58 n=22; DKO: 76.72±5.42 n=25; DKO+Vti1a: 143.52±16.51 n=19, DKO+Vti1b: 173.62±2.90, n=24; Kruskal-Wallis). (F) Percentage histogram of synaptic levels of SNAP25. (G) The synaptic levels of Munc13-1 are rescued by expression of Vti1a or Vti1b at DIV10 (DHZ: 107.49±4.61 n=22; DKO: 61.91±3.32 n=25; DKO+Vti1a: 94.24±6.68 n=19, DKO+Vti1b: 104.98±5.32, n =24; Kruskal-Wallis). (H) Percentage histogram of synaptic levels of Munc13-1. Bars show mean±SEM. Scatterplots and columns represent individual neurons and independent litters, respectively. **p<0.01; ***p<0.001.

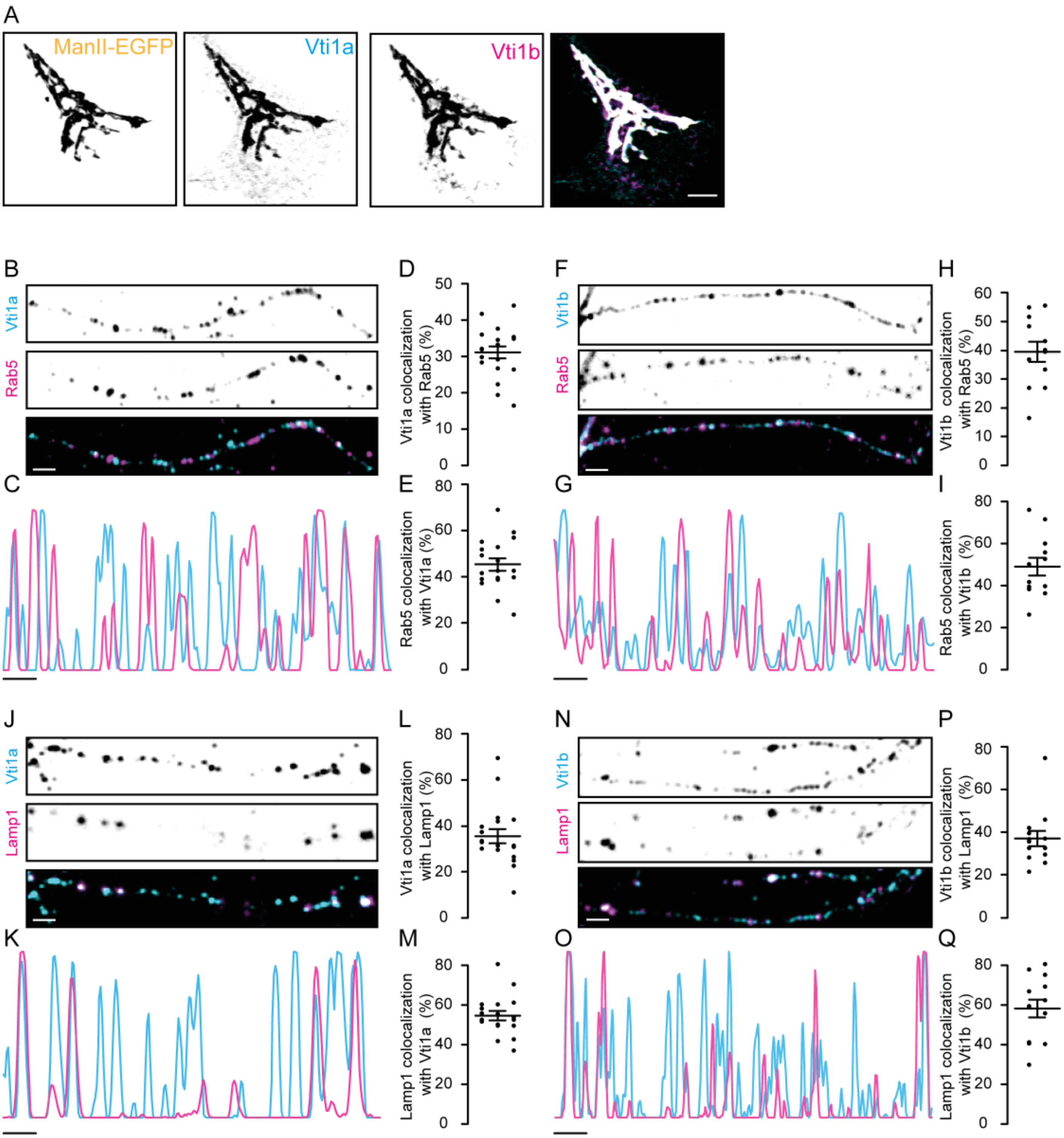

Supplementary Figure 7. Preferential location of Vti1a and Vti1b to the Golgi. (A) Representative examples of DIV-14 wild type neurons expressing the Golgi marker ManII-EGFP and immunostained for Vti1a and Vti1b. (B) Representative examples of DIV-14 wild type neurons immunostained for Vti1a and Rab5. (C) Representative traces from (B) showing partial colocalization of Vti1a and Rab5. (D) Percentage of colocalization of Vti1a with Rab5 (31.11±1.70). (E) Percentage of colocalization of Rab5 with Vti1a (45.30±2.60). (F) Representative examples of DIV-14 wild type neurons immunostained for Vti1b and Rab5. (G) Representative traces from (F) showing partial colocalization of Vti1b and Rab5. (H) Percentage of colocalization of Vti1b with Rab5 (39.52±3.51). (I) Percentage of colocalization of Rab5 with Vti1b (48.93±4.31). (J) Representative examples of DIV-14 wild type neurons immunostained for Vti1a and Lamp1. (K) Representative traces from (J) showing partial colocalization of Vti1a and Lamp1. (L) Percentage of colocalization of Vti1a with Lamp1 (35.52±3.16). (M) Percentage of colocalization of Lamp1 with Vti1a (54.64±2.40). (N) Representative examples of DIV-14 wild type neurons immunostained for Vti1b and Lamp1. (O) Representative traces from (N) showing partial colocalization of Vti1b and Lamp1. (P) Percentage of colocalization of Vti1b with Lamp1 (37.03±3.64). (Q) Percentage of colocalization of Lamp1 with Vti1b (58.24±4.47). Bars show mean±SEM. Scatterplots and columns represent individual neurons and independent litters, respectively. Scale bars=5 μm (A), 2 μm (B-O).

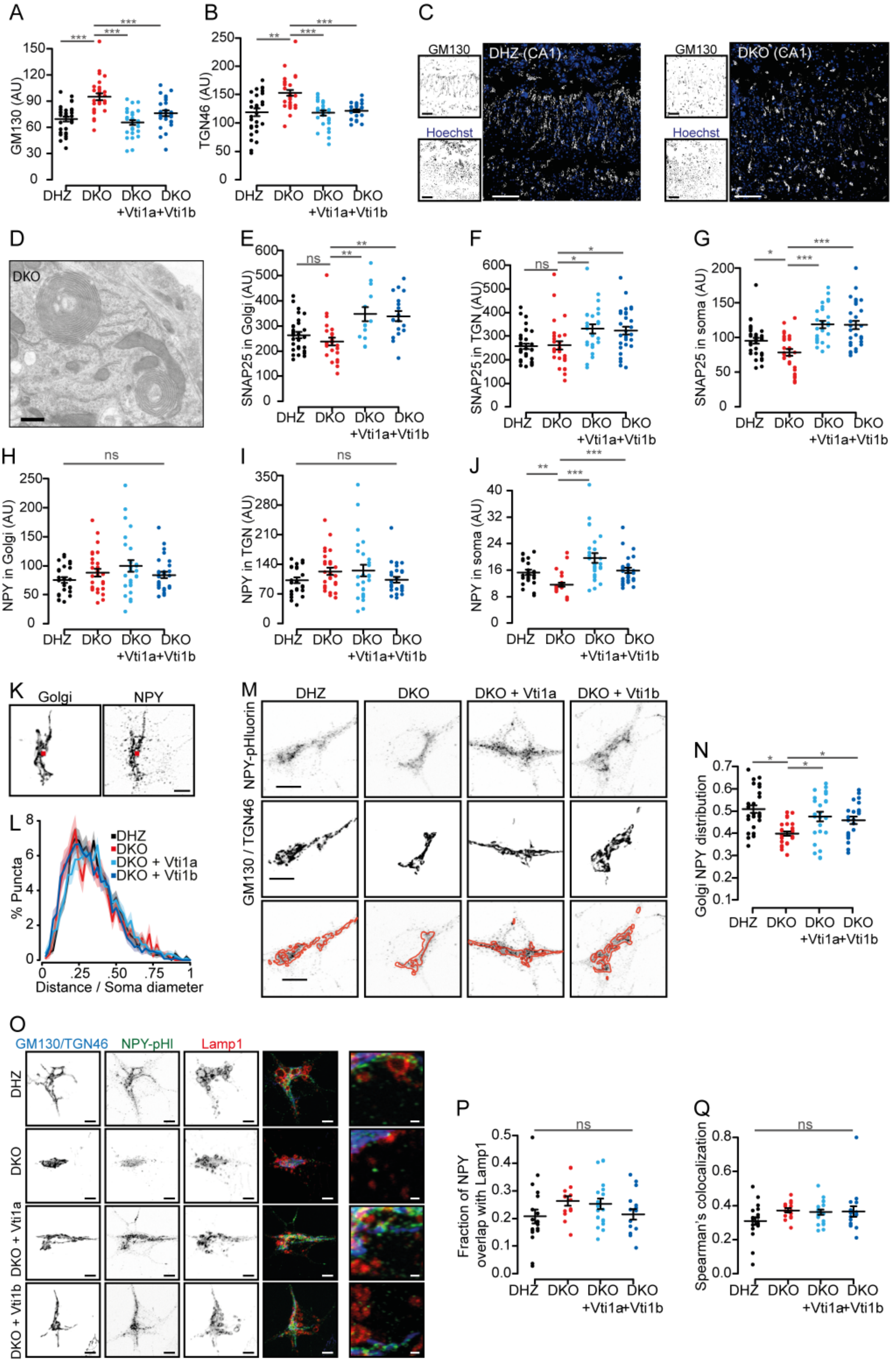

(Legend on next page)

Supplementary Figure 8. Vti1a/b deficient neurons present Golgi abnormalities. (A) GM130 intensity is increased in DKO neurons (DHZ: 69.47±2.92, n=30; DKO: 95.00±3.74, n=29; DKO+Vti1a: 65.57±2.7, n=29; DKO+Vti1b: 73.12±3.22, n=27; one-way ANOVA). (B) TGN46 intensity is increased in DKO neurons (DHZ: 118.79±6.52, n=30; DKO: 152.45±5.39, n=29; DKO+Vti1a: 117.45±4.40, n=29; DKO+Vti1b: 121.17±2.70, n=27; Kruskal-Wallis). (C) Representative sagittal sections of CA1 hippocampal regions at E18.5 stained for GM130 and incubated with Hoechst to visualize nuclei. (D) Example of multilayer membrane structure in DIV-14 DKO neurons. (E) SNAP25 intensity in the MannII-ECFP-positive areas (DHZ: 263.63±11.91, n=31; DKO: 238.42±15.46, n=26; DKO+Vti1a: 347.14±28.16, n=14; DKO+Vti1b: 338.91±19.93 AU, n=18; Kruskal-Wallis). (F) SNAP25 intensity in TGN46-positive areas (DHZ: 256.69±11.76, n=33; DKO: 260.91±17.79, n=29; DKO+Vti1a: 330.41±18.67, n=26; DKO+Vti1b: 323.01±17.33 AU, n=29; Kruskal-Wallis). (G) SNAP25 intensity in the soma, excluding MannII-ECFP and TGN46-positive areas (DHZ: 94.90±4.04, n=33; DKO: 78.23±4.63, n=29; DKO+Vti1a: 118.44±5.13, n=26; DKO+Vti1b: 117.69±6.08 AU, n=29; Kruskal-Wallis). (H) NPY-pHluorin intensity in the GM130-positive areas (DHZ: 75.63±5.03, n=25; DKO: 87.73±6.64, n=28; DKO+Vti1a: 99.49±9.92, n=27; DKO+Vti1b: 84.11±5.53 AU, n=28; Kruskal-Wallis). (I) NPY-pHluorin intensity in TGN46-positive areas (DHZ: 102.39±6.67, n=25; DKO: 123.43±8.50, n=28; DKO+Vti1a: 124.94±13.73, n=27; DKO+Vti1b: 103.33±6.72 AU, n=28; Kruskal-Wallis). (J) NPY-pHluorin intensity in the soma, excluding GM130- and TGN46-positive areas (DHZ: 15.31±0.74, n=25; DKO: 11.56±0.62, n=28; DKO+Vti1a: 19.65±1.41, n=27; DKO+Vti1b: 15.88±0.79 AU, n=28; Kruskal-Wallis). (K) Representative example of a single DHZ neuron expressing NPY-pHluorin and immunostained for GM130 and TGN46. Red dot indicates the centroid of the Golgi-TGN mask. (L) Percentage of NPY-pHluorin per distance to the centroid of the GM130 / TGN46-positive area normalized to the Feret's diameter (longest distance between any two points) of the soma. (M) Representative examples of single DIV-14 neurons expressing NPY-pHluorin and immunostained for GM130 and TGN46. Red lines represent GM130/TGN46-positive areas. (N) The distribution of NPY-pHluorin within GM130/TGN46-positive areas, measured as standard deviation over average normalized NPY-pHIluorin intensity, is lower in DKO neurons (DHZ: 0.48±0.02, n=35; DKO: 0.39±0.01, n=34; DKO+Vti1a: 0.46±0.02, n=33; DKO+Vti1b: 0.45±0.02, n=30; Kruskal-Wallis). (O) Representative examples and zooms of single DIV-5 neurons expressing NPY-pHluorin and immunostained for GM130, TGN46 and the lysosomal marker Lamp1. (P) Fraction of somatic NPY-pHluorin that co-localizes with Lamp1 outside of GM130 / TGN46-positive areas (DHZ: 0.21±0.03, n=19; DKO: 0.26±0.02, n=15; DKO+Vti1a: 0.25±0.02, n=20; DKO+Vti1b: 0.25±0.02, n=17; one-way ANOVA). (Q) Spearman's co-localization coefficient for Lamp1 and NPY-pHluorin outside of GM130- and TGN46-positive areas (DHZ: 0.31±0.02, n=19; DKO: 0.37±0.01, n=15; DKO+Vti1a : 0.36±0.02, n=20; DKO+Vti1b: 0.37±0.03, n=17; Kruskal-Wallis). Bars show mean±SEM. Scatterplots and columns represent individual neurons and independent litters, respectively.*p<0.05; **p<0.01; ***p<0.001. Shaded regions in L and N represent SEM. Scale bars=20 μm (C), 500 nm (D), 5 μm (M, O).

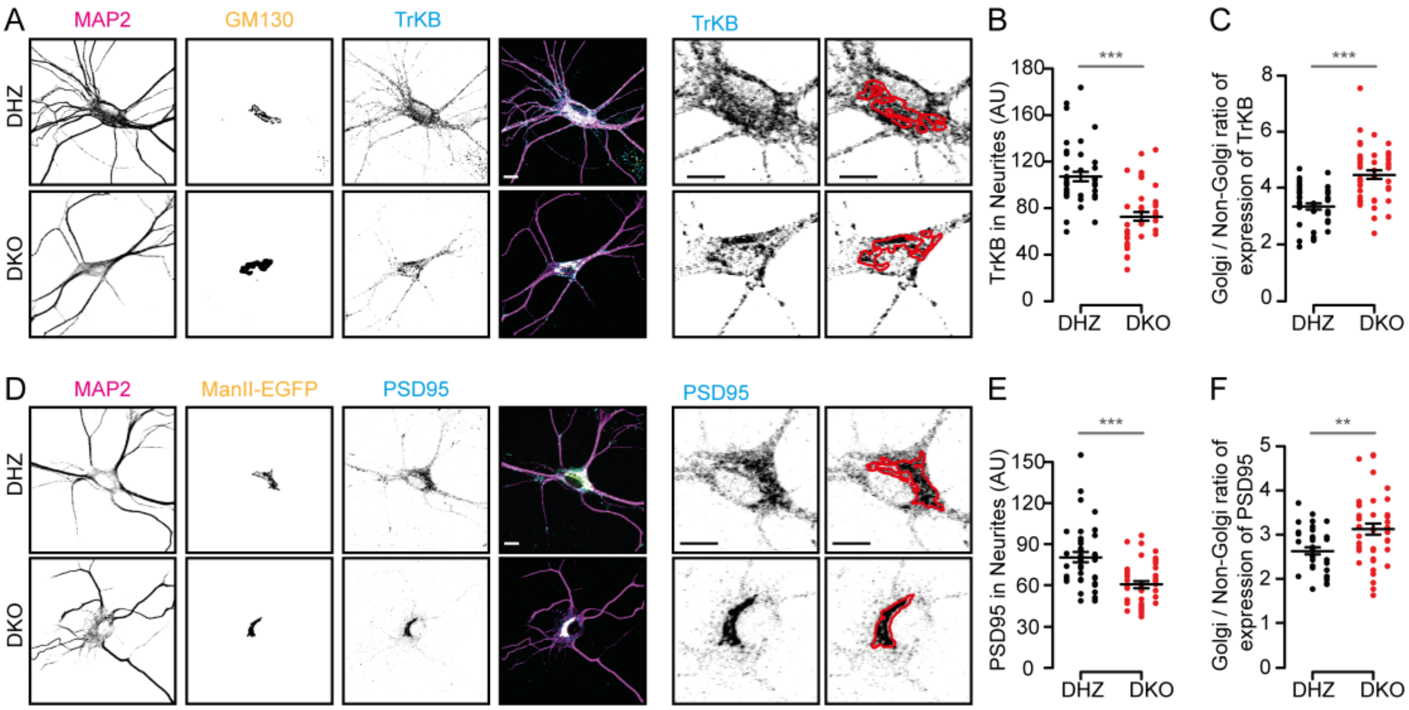

Supplementary Figure 9. Vti1a/b DKO neurons accumulate post-synaptic proteins at the Golgi. (A) Representative examples and zooms of DIV-14 neurons immunostained for MAP2, GM130 and TrKB. (B) Higher immunoreactivity for TrKB receptors in neurites in DHZ (107.11±4.00) than DKO neurons (72.58±3.87 AU; Mann-Whitney). (C) The Golgi-to-non Golgi expression ratio of TrKB receptors is lower in DHZ (3.34±0.11) than DKO neurons (4.46±0.16; t-test). (D) Representative examples and zooms of DIV-14 neurons immunostained for MAP2, GM130 and PSD95. (E) Higher immunoreactivity for PSD95 in neurites in DHZ (80.47±3.74) than DKO neurons (60.50±2.50 AU; Mann-Whitney). (F) The Golgi-to-non Golgi expression ration of PSD95 is lower in DHZ (2.63±0.08) than DKO neurons (3.12±0.12; Mann-Whitney). Bars show mean±SEM. Scatterplots and columns represent individual neurons and independent litters, respectively. **p<0.01; ***p<0.001. Scale bars=10 μm.

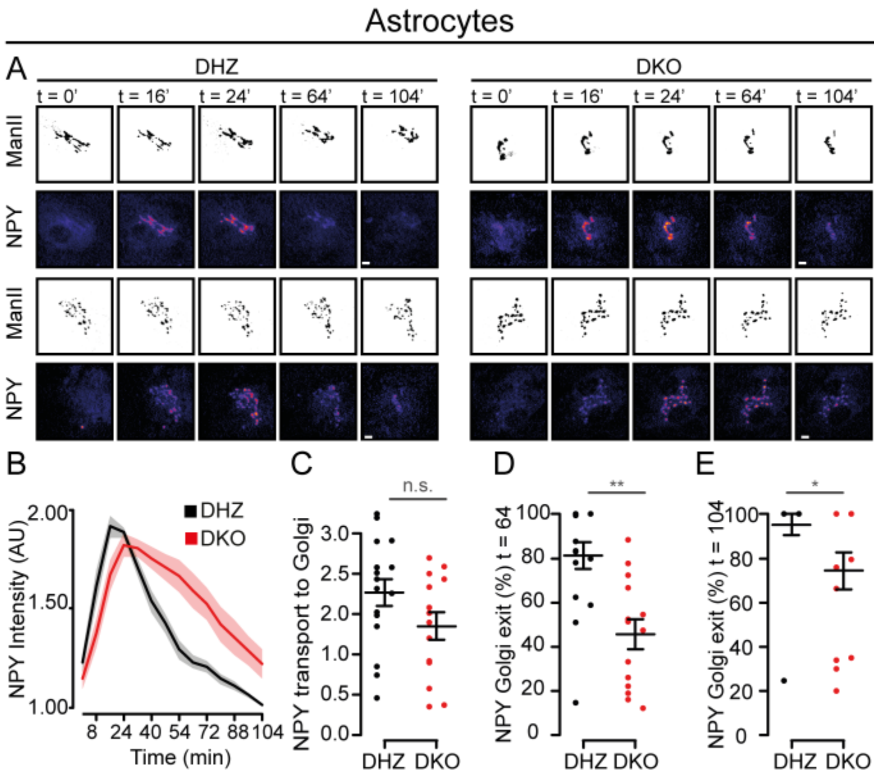

Supplementary Figure 10. Vti1a/b DKO astrocytes present similar cellular phenotypes than neurons. (A) Representative time point examples of astrocytes expressing NPY-SBP-EGFP and ManII-ECFP. Biotin was added at t=0. (B) Intensity of NPY-SBP-EGFP in the Golgi. (C) ER to Golgi transport of NPY, measured as the fitted slope, is similar between groups (DHZ: 2.13±0.20, n =16; DKO: 1.63±0.21, n=14; t-test). (D) Golgi exit of NPY, measured as the percentage of cargo that leaves the Golgi relative to the peak, at t=64 (DHZ: 81.16±6.02, n=16; DKO: 46.71±6.61%, n=14; Mann-Whitney). (E) Golgi exit of NPY, measured as the percentage of cargo that leaves the Golgi relative to the peak, at t=104 (DHZ: 95.30±4.70, n=16; DKO: 74.38±8.37%, n=14; Mann-Whitney). Bars show mean±SEM. Scatterplots and columns represent individual neurons and independent litters, respectively. *p<0.05; **p<0.01; ***p<0.001. Scale bars=20 μm (A), 5 μm (D, I).

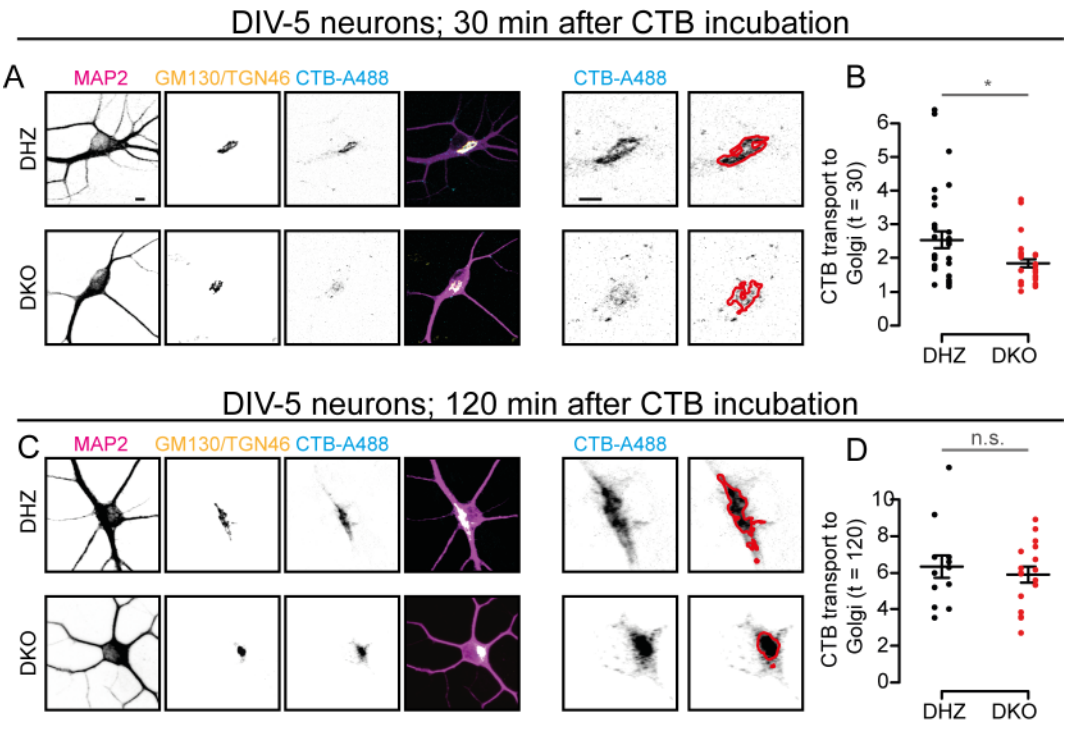

Supplementary Figure 11. Cholera Toxin retrograde transport to the Golgi is affected in DIV-5 Vti1a/b DKO neurons. (A, C) Representative examples and zooms of DIV-5 neurons incubated with CTB-A488 and imaged 30 (A) or 120 minutes (C) after incubation, and immunostained for MAP2, GM130 and TGN46. Red lines represent GM130 / TGN46-positive areas. (B, D) CTB-A488 transport to the Golgi at DIV-5, measured as intensity at the Golgi over rest of the neuron, is reduced in DKO neurons 30 minutes (B) after incubation (DHZ: 2.54±0.25, n=32; DKO: 1.85±0.12, n=31) but not 120 minutes (D) after incubation (DHZ: 6.35±0.62, n=13; DKO: 5.44±0.43, n=17). Bars show mean±SEM. Scatterplots and columns represent individual neurons and independent litters, respectively. *p<0.05. Scale bars =5 μm.

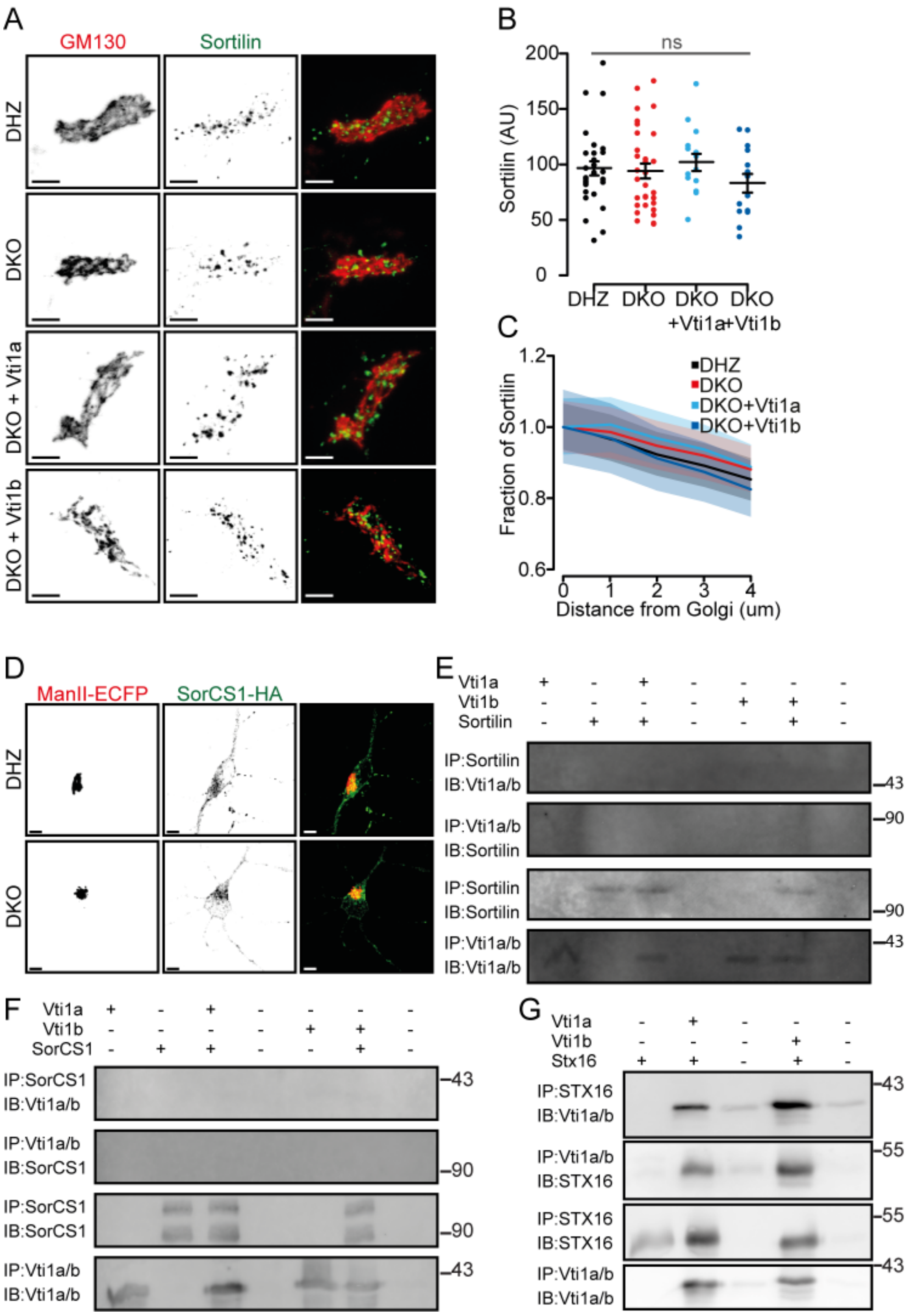

Supplementary Figure 12. Similar levels and distribution of VPS10 protein in Vti1a/b deficient neurons. (A) Representative examples of DIV-14 neurons immunostained for MAP2, GM130 and Sortilin. (B) Sortilin expression in the Golgi is not affected in DKO neurons (DHZ: 96.64± 6.31, n=30; DKO: 94.58±6.72, n=31; DKO+Vti1a: 102.07±7.92, n=15; DKO+Vti1b: 83.27±8.55, n=14; Kruskal-Wallis). (C) Sortilin expression as a distance to the Golgi is similar between groups. Shaded region represents SEM. (D) Representative examples of DIV-14 neurons expressing ManII-ECFP and SorCS1-HA. (E) IP of Vti1a or Vti1b (myc tagged) and flag-tagged Sortilin. (F) IP of Vti1a or Vti1b (myc tagged) and flag-tagged SorCS1.
(G) IP of Vti1a or Vti1b (myc tagged) and flag-tagged Syntaxin-16 (Stx16). Bars show mean±SEM. Scatterplots and columns represent individual neurons and independent litters, respectively. Scale bars =5 µm.

	Area MannII-ECFP (µm^2)	Area GM130 (µm^2)	Area TGN46 (µm^2)
DHZ	35.61 ± 2.96 (31 / 3)	42.96 ± 3.59 (25 / 3)	33.52 ± 2.18 (58 / 6)
DKO	21.18 ± 2.41 (26 / 3)	29.31 ± 2.40 (28 / 3)	22.61 ± 2.10 (57 / 6)
DKO + Vti1a	52.8 ± 6.09 (14 / 3)	44.06 ± 3.49 (27 / 3)	40.07 ± 2.64 (51 / 6)
DKO + Vti1b	44.87 ± 4.46 (18 / 3)	45.77 ± 2.86 (28 / 3)	42.31 ± 2.85 (54 / 6)

Supplementary Table 1. **Golgi area at DIV-5.** Data represents mean ± SEM. The number of observation and independent litters appears in brackets.
