# Supplementary material for: Vti1a/b regulate synaptic vesicle and dense core vesicle secretion via protein sorting at the Golgi
Source: Nat Commun. 2018 Aug 24;9:3421. doi: 10.1038/s41467-018-05699-z (PMC6109172; doi:10.1038/s41467-018-05699-z)
Supplement: Supplementary file 3 — Description of Additional Supplementary Files [file 41467_2018_5699_MOESM3_ESM.pdf]

Description of Additional Supplementary Files

Supplementary Movie 1: Fusion of Synaptophysin-pHluorin-labelled synaptic vesicles upon field stimulation (100 action potentials at 40 Hz). Final NH4+ superfusion dequenches pHluorin and renders the labelled pool of synaptic vesicles visible. Scale bar = 2 µm.

Supplementary Movie 2: Fusion of individual NPY-pHluorin-labelled dense core vesicles upon high frequency stimulation (16 trains of 50 action potentials at 50 Hz). Final superfusion with NH4+ dequenches pHluorin and renders the labelled pool of dense core vesicles visible. Scale bar = 5 µm.
